# ﻿A new species of the genus *Cephalodella* (Rotifera, Monogononta) from Korea, with reports of four additional cephalodellid species

**DOI:** 10.3897/zookeys.1141.91147

**Published:** 2023-01-23

**Authors:** Hee-Min Yang, Gi-Sik Min

**Affiliations:** 1 Department of Biological Sciences and Bioengineering, Inha University, Incheon 22212, Republic of Korea Inha University Incheon Republic of Korea

**Keywords:** COI, morphology, new records, Notommatidae, rotifers, SEM, taxonomy

## Abstract

A new monogonont rotifer, *Cephalodellabinoculata***sp. nov.**, was described from a soil sample collected in Korea. The new species is morphologically similar to *C.carina* but is distinguished by having two frontal eyespots, a vitellarium with eight nuclei, and the shape of its fulcrum. We also described four other cephalodellid species collected in Korea; *Cephalodellaauriculata*, *C.catellina*, *C.gracilis*, and *C.tinca*. Of these four species, *C.gracilis* and *C.tinca* were newly recorded in Korea. We provided the morphological characteristics of the five *Cephalodella* species along with photographs of trophi observed with a scanning electron microscope. Furthermore, we provided the mitochondrial cytochrome *c* oxidase subunit I gene sequences of the five species.

## ﻿Introduction

The genus *Cephalodella* Bory de St. Vincent, 1826 is one of the most species-rich taxa in the phylum Rotifera Cuvier, 1817, containing 171 species worldwide (Segers 2007; Jersabek and Leitner 2013). This taxon is easily found in various environments but is difficult to distinguish due to its morphological similarity and fragile external characteristics (Jersabek et al. 2011). Like other taxa of Rotifera, *Cephalodella* has been mainly studied in Europe, and their biology, ecology, and variability are not well known because of the lack of research (Nogrady and Pourriot 1995).

In Korea, a total of seven cephalodellid species have been recorded: *Cephalodellaauriculata* (Müller, 1773), *C.catellina* (Müller, 1786), *C.forficula* (Ehrenberg, 1838), *C.gibba* (Ehrenberg, 1830), *C.hoodii* (Gosse, 1886), *C.innesi* Myers, 1924, and *C.ventripes* (Dixon-Nuttall, 1901) (Yamamoto 1953; Turner 1986; Song and Jin 2000; Song 2014; Song 2018; National Institute of Biological Resources 2022). Including the genus *Cephalodella*, studies on the species diversity of the family Notommatidae Hudson & Gosse, 1886 in Korea are insufficient. More than 250 species of notommatid rotifers have been recorded worldwide, but in Korea, only 16 species have been recorded so far (Song and Jin 2000; National Institute of Biological Resources 2022). Korea has a diverse climate and habitat compared to its territorial size; thus, it is expected that many notommatid rotifers will be discovered through continuous study (Republic of Korea 2014).

In this study, we identified five cephalodellid rotifers, one of which was a new species. Two species, *Cephalodellagracilis* (Ehrenberg, 1830) and *C.tinca* Wulfert, 1937 were newly recorded in Korea and two others, *C.auriculata* and *C.catellina*, have previously been recorded in Korea. However, since the first reported paper on rotifers in Korea (Turner, 1986) did not include descriptions for these two species, we have described the two Korean specimens in this study. Here, we provide the morphological characteristics of the five species along with the photographs of trophi observed with scanning electron microscope (SEM). In addition, we deciphered the mitochondrial cytochrome *c* oxidase subunit I (COI) gene sequences of the five species.

## ﻿Materials and methods

**Figure 1.**
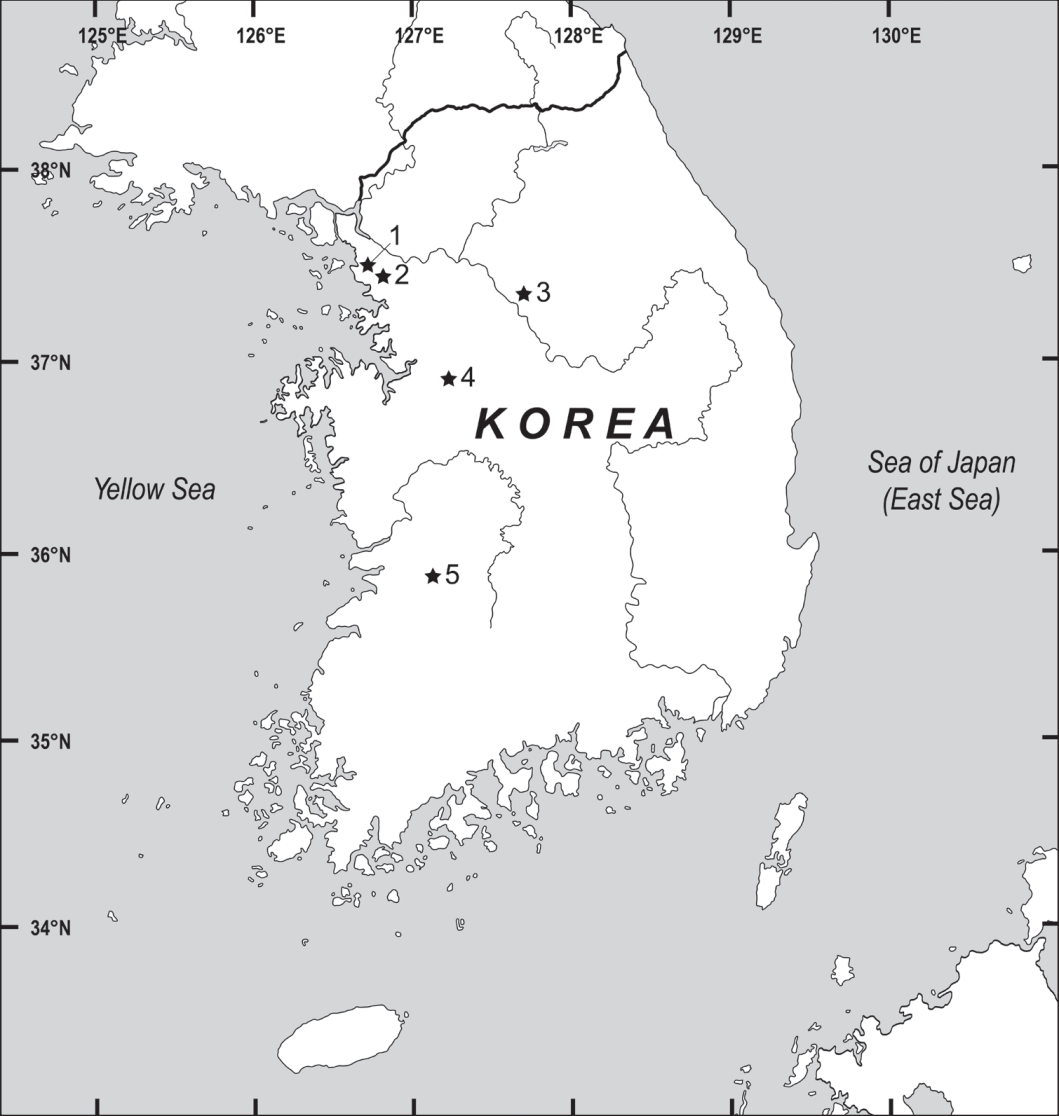
Map showing the collection sites of the rotifers in this study **1***Cephalodellaauriculata* (Müller, 1773) **2***C.binoculata* sp. nov. **3***C.tinca* Wulfert, 1937 **4***C.gracilis* (Ehrenberg, 1830) **5***C.catellina* (Müller, 1786).

Specimens were collected and isolated from a pond, reservoir and soil samples (Fig. 1). The rotifers inhabiting pond and reservoir were collected using a 50-µm mesh plankton net and transferred to the laboratory alive. In case of soil sample treatment, we dried the soil samples at room temperature for several weeks and rewetted them using mineral water in a plant culture dish (310100, SPL Life Science, Korea). After hatching of the rotifers, they were isolated in a new plant culture dish under a stereo microscope (SZX7, Olympus, Japan) and stored in an incubator at 20 °C. Before the observation and preservation of living rotifers, a few drops of 1% bupivacaine solution (B5274, Sigma-Aldrich, USA) were used for anesthesia. The specimens were then observed under an optical microscope (DM2500, Leica, Germany) at magnifications of ×400–1000. Photographs and videos of the specimens were obtained using a digital camera (EOS 6D Mark II, Canon, Japan) mounted on an optical microscope. Trophi were isolated using commercial bleach containing 4–5% NaClO (Yuhan-Clorox, Korea) and prepared for SEM following the methods of De Smet (1998). Two SEM instruments, SU8010 and S-4300SE (Hitachi, Japan), were used for observation at an accelerating voltage of 7–10 kV. External characteristics and trophi elements were measured using ImageJ 1.53k (https://imagej.nih.gov/ij/) (Abràmoff et al. 2004).

Morphological identification of rotifers was based on descriptions of Koste (1978), Nogrady and Pourriot (1995), and Jersabek and Leitner (2013). All specimens described in this study were deposited at the National Institute of Biological Resources (NIBR), Korea.

Genomic DNA was extracted using a LaboPass^TM^ Tissue Genomic DNA Isolation Kit Mini (Cosmo Genetech, Korea). Partial COI gene was amplified using the 30F/885R primers (Zhang et al. 2021). The PCR amplifications were conducted in a final volume of 25µL under the following conditions: 2 min at 95 °C for the initial denaturation, followed by 40 cycles of 95 °C for 15 s, 51 °C for 30 s, 72 °C for 1 min, and a final extension at 72 °C for 5 min. In the case of *C.auriculata* and *C.tinca*, the primer sets mlCOIintF/jgHCO2198 (Leray et al. 2013) and LCO1490/HCO2198 (Folmer et al. 1994) were used at an annealing temperature of 45 °C. PCR products were visualized by 1% agarose gel electrophoresis, and purified using a LaboPass^TM^ PCR Purification Kit (Cosmo Genetech). DNA sequencing was performed at Macrogen (Korea), and the sequences were trimmed and aligned using Geneious ver. 8.1.9 (https://www.geneious.com). Genetic distance was calculated using MEGA ver. 11 with the Kimura 2-parameter model (K2P) (Tamura et al. 2021). All the extracted DNAs of the five species were deposited at the NIBR, and COI sequences were uploaded to GenBank.

The maximum-likelihood (ML) tree was inferred based on the partial COI gene sequences of 11 notommatid species and one euchlanid species (Table 1). The ML tree was constructed using IQ-TREE ver. 1.6.12, with the GTR+I+G model and 1000 replicates (Nguyen et al. 2015; Kalyaanamoorthy et al. 2017).

**Table 1.** List of species for which COI sequence data was used for molecular analysis.

Family	Species	GenBank No.	Reference
Notommatidae	*Cephalodellabinoculata* sp. nov.	ON898529 (759 bp)	This study
	*Cephalodellaauriculata* (Müller, 1773)	ON898533 (315 bp)	
	*Cephalodellacatellina* (Müller, 1786)	ON898532 (759 bp)	
	*Cephalodellagracilis* (Ehrenberg, 1830)	ON898535 (759 bp)	
	*Cephalodellatinca* Wulfert, 1937	ON898534 (660 bp)	
	Cephalodellacf.gibba (Ehrenberg, 1830)	JX216594 (661 bp)	García-Morales and Elías-Gutiérrez (2013)
	*Eothiniaelongata* (Ehrenberg, 1832)	DQ079964 (660 bp)	Sørensen et al. (2006)
	*Eosphoraehrenbergi* Weber, 1918	HQ444173 (646 bp)	Curini-Galletti et al. (2012)
	*Notommataallantois* Wulfert, 1935	MT521624 (661 bp)	Fontaneto et al. (2021)
	*Notommatacodonella* Harring & Myers, 1924	DQ297785 (660 bp)	Sørensen and Giribet (2006)
	*Pleurotrochapetromyzon* Ehrenberg, 1830	EU499803 (583 bp)	Swanstrom et al. (2011)
Euchlanidae (Outgroup)	*Euchlanisdilatata* Ehrenberg, 1830	JX216599 (661 bp)	García-Morales and Elías-Gutiérrez (2013)

## ﻿Results and discussion

In the present study, we identified five cephalodellid species in Korea; *C.auriculata*, *C.binoculata* sp. nov., *C.catellina*, *C.gracilis*, and *C.tinca*. The new species, *C.binoculata* sp. nov., was distinguished from other cephalodellid species by a combination of the following characteristics: two distinct frontal eyespots, short tail and toes, vitellarium with eight nuclei, and the shape of the trophi components. Two species, *C.gracilis* and *C.tinca* were newly recorded in Korea. *Cephalodellagracilis* is a common species worldwide. However, the morphological characteristics of *C.gracilis* have been reported to exhibit high morphological variation (Nogrady and Pourriot 1995), and it is necessary to re-examine these characteristics through morphological redescription and molecular analysis. *Cephalodellatinca* is probably a cosmopolitan species and has been recorded in the Australian, Neotropical, Oriental, and Palearctic regions (Segers 2007). The remaining two species, *C.auriculata* and *C.catellina* were recorded in Korea by Turner (1986) as a species list without description. Therefore, we described the Korean specimens of the two species and provided photographs of the trophi observed using SEM.

In this study, we obtained partial COI sequences from each of the five species and constructed an ML tree using the sequences of 11 notommatid rotifers and one euchlanid rotifer. The sequence of *Euchlanisdilatata* Ehrenberg, 1830 was used as the outgroup. The final length of the sequence alignment was 561 bp, and the genetic distance between the notommatid species was 0.172–0.412 (Table 2). The species in the genus *Cephalodella* formed a monophyletic group, with a support value of 100 (Fig. 7). The new species, *C.binoculata* sp. nov., formed a clade with *C.auriculata* and *C.gracilis* and was located closest to *C.auriculata*. However, the phylogenetic relationships between species within the *Cephalodella* was not clearly revealed when compared using morphological characteristics. Although more than 170 morphospecies of the genus *Cephalodella* have been recorded worldwide (Nogrady and Pourriot 1995; Segers 2007; Jersabek and Leitner 2013), only seven sequences from two species, *C.forficula* and *C.gibba*, have been registered in GenBank. For the phylogenetic study of cephalodellid rotifer species, further acquisition and analysis of the COI sequences and nuclear gene sequences such as 18S ribosomal RNA or internal transcribed spacer (ITS), is required.

**Table 2.** Genetic distance of notommatid species and outgroup (K2P distance).

Species	GenBank No.	1	2	3	4	5	6	7	8	9	10
*Cephalodellabinoculata* sp. nov.	ON898529										
*Cephalodellaauriculata*	ON898533	0.251									
*Cephalodellacatellina*	ON898532	0.274	0.335								
*Cephalodellagracilis*	ON898535	0.229	0.312	0.282							
*Cephalodellatinca*	ON898534	0.243	0.321	0.267	0.293						
Cephalodellacf.gibba	JX216594	0.297	0.412	0.323	0.289	0.293					
*Eothiniaelongata*	DQ079964	0.293	0.374	0.349	0.363	0.331	0.373				
*Eosphoraehrenbergi*	HQ444173	0.321	0.386	0.327	0.306	0.319	0.351	0.309			
*Notommataallantois*	MT521624	0.235	0.385	0.312	0.307	0.296	0.340	0.262	0.207		
*Notommatacodonella*	DQ297785	0.237	0.347	0.324	0.313	0.283	0.346	0.310	0.229	0.172	
*Pleurotrochapetromyzon*	EU499803	0.327	0.369	0.364	0.345	0.374	0.363	0.303	0.301	0.278	0.317

### ﻿Systematic account

**Phylum Rotifera Cuvier, 1817**

**Class Eurotatoria De Ridder, 1957**

**Subclass Monogononta Plate, 1889**

**Order Ploima Hudson & Gosse, 1886**

**Family Notommatidae Hudson & Gosse, 1886**

#### Genus *Cephalodella* Bory de St. Vincent, 1826

##### ﻿ Cephalodella binoculata sp. nov.

Taxon classificationAnimaliaPloimaNotommatidae

4A3D4827-6C19-589F-B4A2-DB8CC61CA71A

https://zoobank.org/D9C8E9C8-55AD-4E49-A7A7-713E3B413D78

###### Material examined.

**Type locality.** Soil from Incheon, Republic of Korea (37°24.788'N, 126°44.738'E), 19 Jun. 2019, Kyu-Seok Chae leg. ***Holotype***. 1 female, glycerol permanent slide, NIBRIV0000896982. ***Paratype***. 2 female, glycerol permanent slides, NIBRIV0000896983, NIBRIV0000896984; trophi preparation for SEM, NIBRIV0000896985.

###### Differential diagnosis.

*Cephalodellabinoculata* sp. nov. was most similar to *C.carina* Wulfert, 1959 in terms of frontal eyes, type B virgate trophi, dorsally curved toes, total length/toe length ratio, and short tail. The new species, however, was distinguished from *C.carina* by the following characteristics: (1) the new species has two distinct eyespots, whereas *C.carina* has one small eyespot; (2) the vitellarium of the new species contains eight nuclei, while that of *C.carina* contains six; and (3) the fulcrum of the new species is straight and without extension at the distal end, while the fulcrum of *C.carina* is thicker at the distal end.

The new species also resembles *C.gibboides* Wulfert, 1951 and *C.graciosa* Wulfert, 1956. However, it is distinguished from *C.gibboides* by the shape of its manubrium and tail length. The manubrium of *C.gibboides* has a bump in the middle with no basal lamellae, whereas the new species has basal lamellae in the manubrium and no bumps in the middle. The shape of the distal end of the manubrium also differed between the two species. The tail of *C.gibboides* covers the foot, whereas that of the new species is short. The new species is distinguished from *C.graciosa* in several morphological characteristics as follows: (1) the trophi of the new species is symmetrical, while that of *C.graciosa* is asymmetrical; (2) the manubrium of the new species has basal lamellae, while that of *C.graciosa* does not; (3) the new species has two eyespots, while *C.graciosa* has one eyespot; and (4) the new species has eight nuclei in the vitellarium, while *C.graciosa* has six.

###### Description.

**Figure 2.**
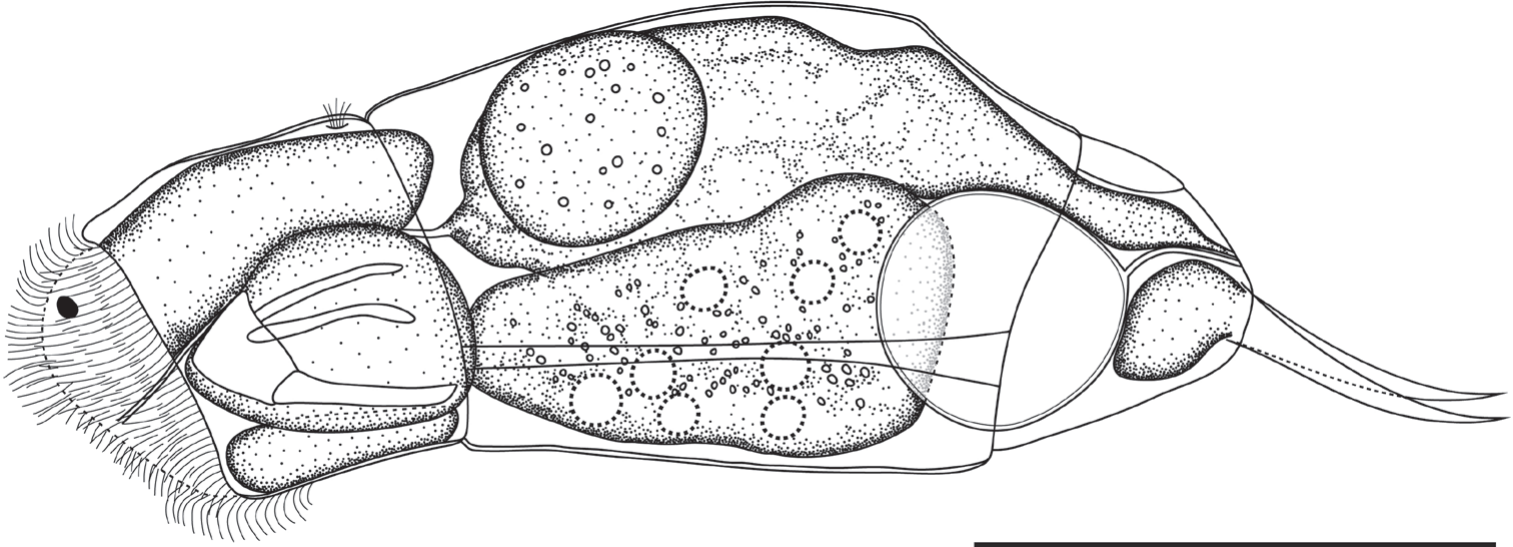
Line drawing of *Cephalodellabinoculata* sp. nov., lateral view. Scale bar: 50 μm.

**Figure 3.**
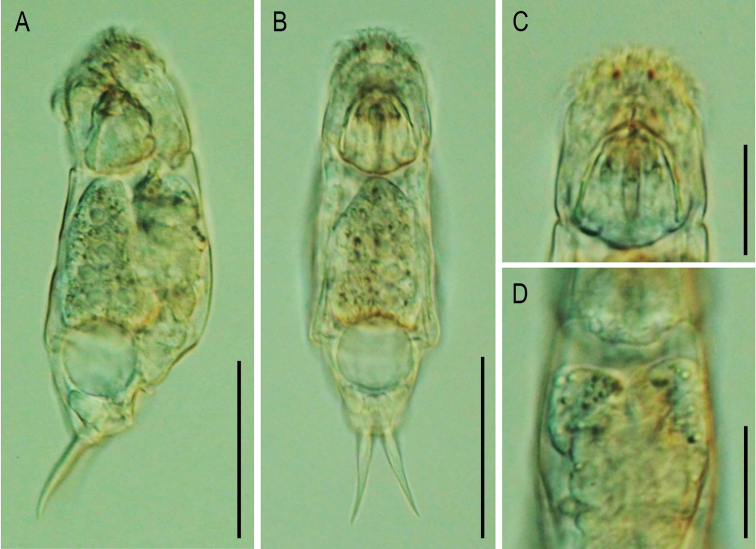
Live specimen of *Cephalodellabinoculata* sp. nov. observed under the optical microscope **A** lateral view **B** ventral view **C** eyespots **D** neck region and gastric glands, dorsal view. Scale bars: 50 μm (**A, B**); 20 μm (**C, D**).

**Figure 4.**
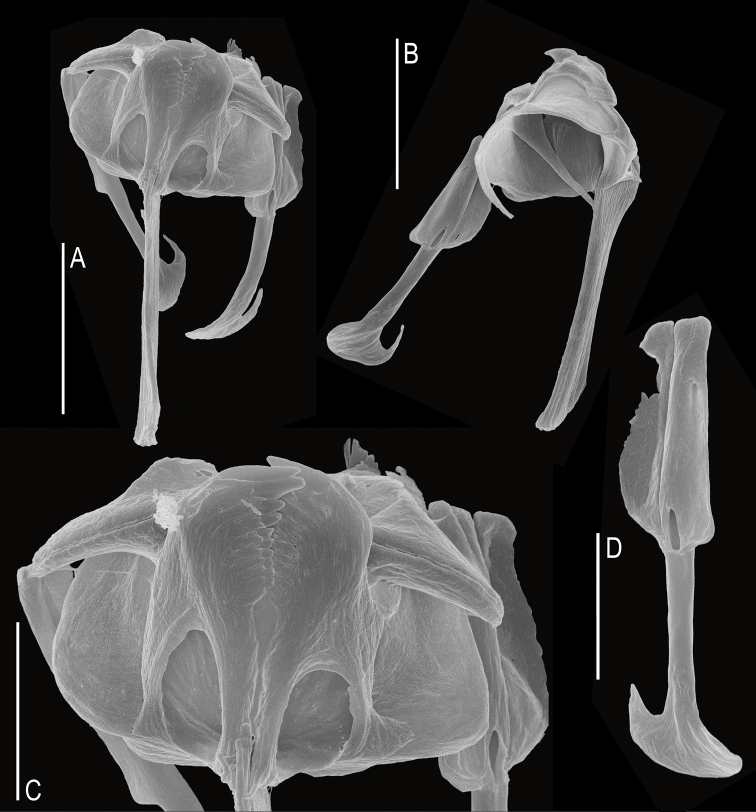
SEM image of the trophi of *Cephalodellabinoculata* sp. nov. **A** ventral view **B** dorsolateral view **C** detail rami and unci, ventral view **D** detail manubrium, lateral view. Scale bars: 10 μm (**A, B**); 5 μm (**C, D**).

**Female.** Body moderately elongated and not laterally compressed (Figs 2, 3A, B). Dorsal and ventral margins slightly convex; posterior third of trunk gradually tapered to the foot. Lorica soft, transparent, and comprised of three body plates. Dorsal and ventral plates separated by narrow lateral sulci. Tail short and rounded. Head large, almost one-quarter of the total length. Head and trunk clearly distinguished by the neck fold. Corona oblique, convex, without lips. Dorsal antenna located near the junction of the head and trunk. Foot trapezoidal shape and moderate size, approximately 15% of the total length. Foot widest at the front and narrowed toward the back. Caudal setae absent. Short tail covered only part of the foot. Toes symmetrical and short, accounting for 16–17% of the total length. Toes smoothly tapered to posterior end, without any spines. In the lateral view, toes curved dorsally. In the dorsal view, toes always curved outwards. Saccate large brain extending over the neck fold. No retrocerebral organ. Two distinct red eyespots located in front of the head (Fig. 3C). Distance between the two eyespots far and clear. Mastax large, with elongated salivary gland. Esophagus thin, passing between the brain and mastax. Gastric glands large, oval shaped, containing round granules, and located in the antero-dorsal part of the stomach (Fig. 3D). Stomach colorless and indistinctly separated from the intestine. Anus located near the posterior end of the foot. Bladder round and large when fully filled. Vitellarium large with eight nuclei. Pedal glands short, sac-shaped. Trophi virgate, type B (see Fischer and Ahlrichs 2011), almost symmetrical (Fig. 4A). Rami with no alulae on posterior end (Fig. 4C). Basal chamber of rami wide, left side relatively larger than right at distal end. Shape of the subbasal chamber foramina also slightly asymmetrical; both foramina oval shaped, but the right foramen larger in length. Inner margin of rami with two distinct teeth and several comb-like teeth (Fig. 4C). Fulcrum long and straight in the ventral view (Fig. 4A). Terminal end of fulcrum simple, without any thickening or expanded shape. In the lateral view, the ventral margin straight and relatively thick (Fig. 4B). No basal apophysis on fulcrum. Uncus with a large, single tooth. Manubria symmetrical. Each manubrium with a basal lamella, the length of which was approximately half of that of the manubrium (Fig. 4D). Middle part of the manubrium with oblong-shaped foramina. Shaft of the manubrium thick and straight in lateral view; while terminal end curved inward in ventral view. Terminal end crutch-shaped, dorsal side stubby, ventral side pointed and curved upwards (Fig. 4B, D).

Characteristics of male and eggs remain unknown.

***Measurement*.** Total length 134–155 μm, toe 26–29 μm, trophi 24–28 μm, ramus 8–9 μm, fulcrum 15–17 μm, manubrium 14–17 μm.

###### Etymology.

The specific name, *binoculata*, derived from the Latin word *bi*, meaning “two” and *oculata*, meaning “eyed”.

###### Molecular data.

Partial COI sequences were obtained from three specimens of *C.binoculata* sp. nov. (NIBR deposit numbers, NIBRGR0000649735–NIBRGR0000649737; GenBank accession numbers, ON898529–ON898531).

##### ﻿ Cephalodella auriculata

Taxon classificationAnimaliaPloimaNotommatidae

(Müller, 1773)

10B680BD-4D06-5DB3-9A00-F0962620DC96

###### Material examined.

Pond in Incheon Metropolitan City, Republic of Korea (37°27.020'N, 126°39.345'E), 2 Dec. 2021, Hee-Min Yang leg. NIBRIV0000896986, 1 female, glycerol permanent slide.

###### Remarks.

**Figure 5.**
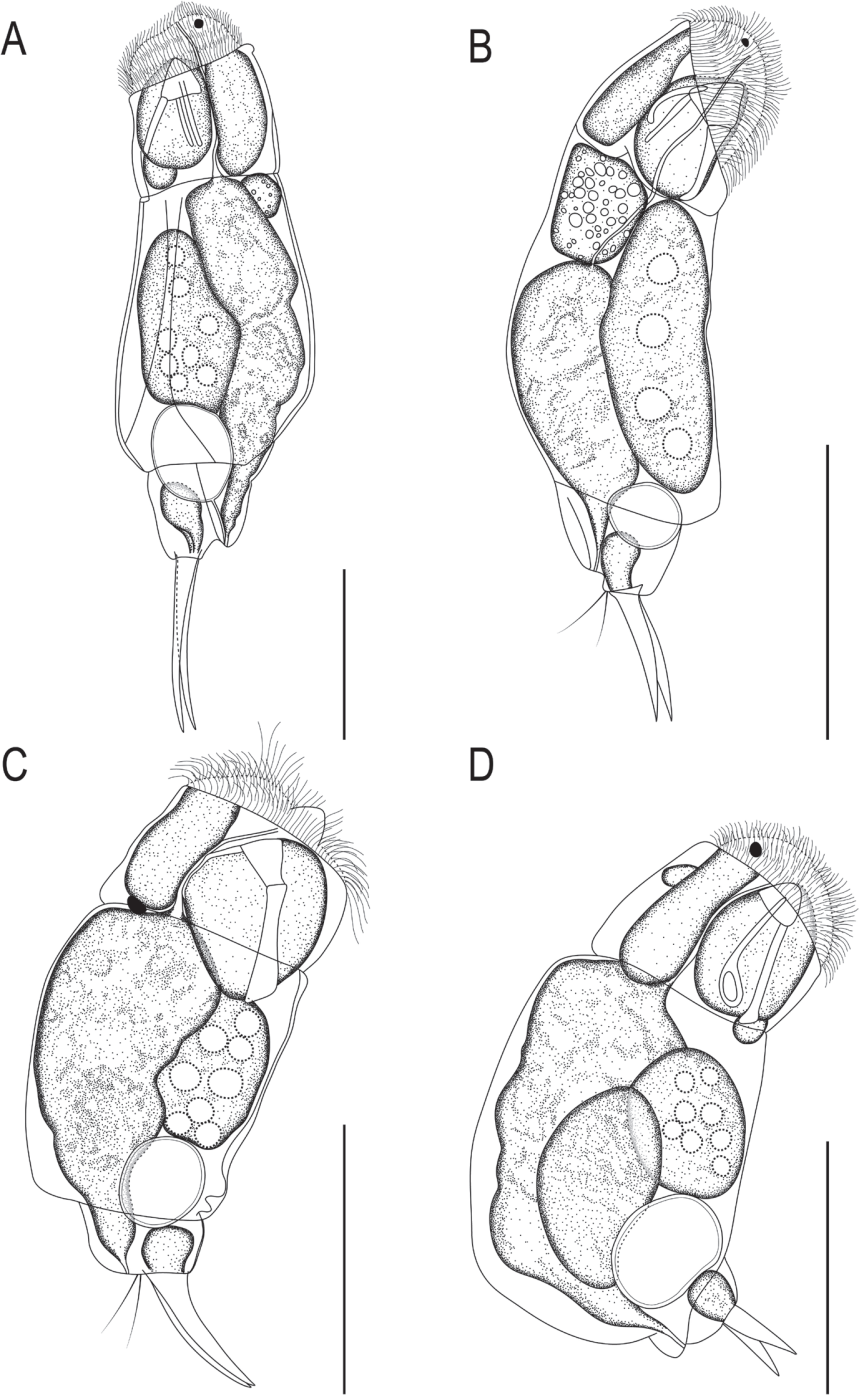
Line drawing of cephalodellid rotifers **A***Cephalodellatinca* Wulfert, 1937 **B***C.gracilis* (Ehrenberg, 1830) **C***C.auriculata* (Müller, 1773) **D***C.catellina* (Müller, 1786). Scale bars: 50 μm.

**Figure 6.**
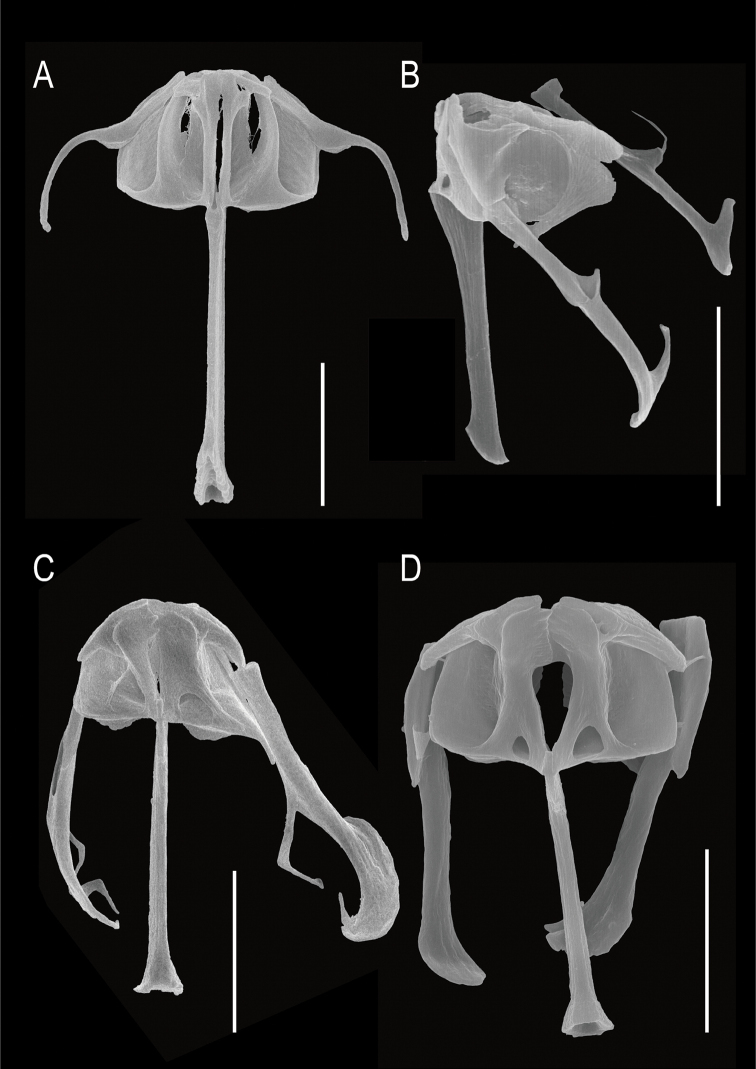
SEM image of the trophi of cephalodellid rotifers **A***Cephalodellaauriculata* (Müller, 1773) **B***C.gracilis* (Ehrenberg, 1830) **C***C.catellina* (Müller, 1786) **D***C.tinca* Wulfert, 1937. Scale bars: 10 μm.

The morphological characteristics of the Korean specimens generally corresponded to those reported in a previous study (Nogrady and Pourriot 1995). The body was soft and stout, 110–130 μm in length (Fig. 5C). The head was large and as wide as the body. Foot was short and wide. The toes were short, 23–28 μm in length. The two toes were equal in length and curved ventrally. One red cerebral eye was located at the posterior end of the saccate brain. The vitellarium had eight nuclei. Trophi was symmetrical and virgate type A, 30 μm in length (Fig. 6A). The fulcrum was long and straight. The manubrium was thin and curved. The rami of Korean specimen had blunt teeth at the apical part, whereas the specimens of previous studies had no teeth at the apical part (Koste and Shiel 1991; Nogrady and Pourriot 1995).

###### Molecular data.

Partial COI sequence was obtained from one Korean specimen (NIBR deposit number, NIBRGR0000649738; GenBank accession number, ON898533).

##### ﻿ Cephalodella catellina

Taxon classificationAnimaliaPloimaNotommatidae

(Müller, 1786)

E08178C4-8995-5D25-8633-6AE437BBEB6F

###### Material examined.

Reservoir in Wanju-gun, Jeollabuk-do, Republic of Korea (35°50.196'N, 127°00.975'E), 27 Mar. 2022, Hee-Min Yang leg. NIBRIV0000896987, 1 female, glycerol permanent slide.

###### Remarks.

Korean specimens of *C.catellina* had morphological characteristics that were generally consistent with those reported in previous studies (Koste and Shiel 1991; Nogrady and Pourriot 1995). The body was short and stout, and 100 μm in length (Fig. 5D). The posterior end of the body bulging. The head was large and approximately one-third of its total length. The foot and toes were located ventrally. The two toes were short and symmetrical, 12–16 μm in length (Fig. 6C). The two frontal eyes were red. The vitellarium had eight nuclei. The salivary glands were located under the mastax. Trophi was asymmetrical, virgate type C, and 25 μm in length. The fulcrum was straight and long, with a slightly expanded distal end. The manubria were asymmetrical and curved inward. The right manubrium was larger than left manubrium. Distal ends of both manubria had incomplete loop. The right ramus had tooth-like alula.

###### Molecular data.

Partial COI sequence was obtained from one Korean specimen (NIBR deposit number, NIBRGR0000649739; GenBank accession number, ON898532).

##### ﻿ Cephalodella gracilis

Taxon classificationAnimaliaPloimaNotommatidae

(Ehrenberg, 1830)

B9479D24-3D27-57C9-A9F4-37070223F3D3

###### Material examined.

Soil from Cheonan-si, Chungcheongnam-do, Republic of Korea (36°54.095'N, 127°12.380'E), 22 Jun. 2019, Hee-Min Yang leg. NIBRIV0000879592, 1 female, glycerol permanent slide.

###### Remarks.

The body size of the Korean specimens was 120–125 μm in length (Fig. 5B). The soft body was elongated and compressed laterally. The head was clearly distinguished from the body by a neck fold. The foot was conical in shape, short, and half the length of the toes. The length of the toes was 20–25 μm, less than one-fifth of the total length. The two toes were equal in length and slightly curved dorsally. One red eye was located at the front of the head. The vitellarium was large and contained four nuclei. The large trophi was symmetrical, virgate type B, and had a length of 20 μm (Fig. 6B). The fulcrum was long and straight without expansion at the end. The manubrium was long and crutched, with a bulge in the middle. The uncus had one tooth and was less than half the length of the manubrium. The rami were denticulated.

*Cephalodellagracilis* has been reported to have high morphological variation in the shape of the toes and trophi. The Korean specimen had dorsally curved toes that gradually tapered toward the end. The trophi shape of the Korean specimen did not correspond to a specific specimen but was most similar to that described by Jersabek et al. (2003) in that it had a straight, slender fulcrum without expansion and a crutched manubrium end. However, this species can be regarded as a species complex, based on its morphological diversity and cosmopolitan distribution. Therefore, it is necessary to re-examine it through morphological redescription and molecular analysis.

###### Molecular data.

Partial COI sequences were obtained from two Korean specimens (NIBR deposit numbers, NIBRGR0000649741, NIBRGR0000649742; GenBank accession numbers, ON898535, ON898536).

##### ﻿ Cephalodella tinca

Taxon classificationAnimaliaPloimaNotommatidae

Wulfert, 1937

189774A0-AD2F-50E3-9B4E-E7072F9ADDE3

###### Material examined.

Soil from Yeoju-si, Gyeonggi-do, Republic of Korea (37°18.483'N, 127°41.067'E), 26 Sep. 2019, Kyu-Seok Chae leg. NIBRIV0000895434, 1 female, glycerol permanent slide.

###### Remarks.

The body was 200–220 μm long, elongated, and laterally compressed (Fig. 5A). The dorsal and ventral margins were slightly bulbous in the lateral view. The lorica was flexible and transparent. The head was large, approximately one-fourth of the total length, and clearly distinguished from the body by the neck fold. The tail was rounded and as long as the foot. The toes were equal in length, slightly curved dorsally, and approximately one-fourth of the total length. A pair of red eyes was located at the front of the head. The mastax was large and had a salivary gland. The gastric glands were round and contained several granules. The vitellarium was large and had eight nuclei. The trophi was virgate type D, and symmetrical (Fig. 6D). The fulcrum was straight and slightly spatulated at the posterior end. The manubrium was thick and had basal lamellae. The tip of the manubrium expanded and curved inward. The uncus had one large tooth.

**Figure 7.**
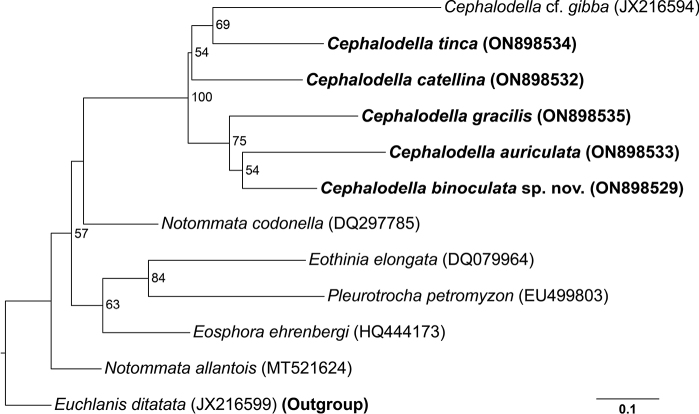
Maximum-likelihood (ML) phylogenetic tree based on COI sequences. Numbers on nodes indicate bootstrap value (BV). Only BV over 50% are shown. Scale bar indicates number of nucleotides substitutions per site.

Morphological characteristics of Korean *C.tinca* specimens corresponded well to the original description except for the size of body length. The body length of the Korean specimen was 200–220 μm, which was slightly smaller than the original description (260–280 μm) (Wulfert 1937).

###### Molecular data.

Partial COI sequence was obtained from one Korean specimen (NIBR deposit number, NIBRGR0000649740; GenBank accession number, ON898534).

## Supplementary Material

XML Treatment for Cephalodella binoculata

XML Treatment for Cephalodella auriculata

XML Treatment for Cephalodella catellina

XML Treatment for Cephalodella gracilis

XML Treatment for Cephalodella tinca
